# Dynamic Crosstalk between Vascular Smooth Muscle Cells and the Aged Extracellular Matrix

**DOI:** 10.3390/ijms221810175

**Published:** 2021-09-21

**Authors:** Joao Carlos Ribeiro-Silva, Patricia Nolasco, Jose Eduardo Krieger, Ayumi Aurea Miyakawa

**Affiliations:** 1 Laboratorio de Genetica e Cardiologia Molecular—LIM-13, Instituto do Coraçao (InCor), Hospital das Clinicas HCFMUSP, Faculdade de Medicina, Universidade de Sao Paulo, Sao Paulo 05403-000, Brazil; ribeiro.jc@usp.br (J.C.R.-S.); j.krieger@hc.fm.usp.br (J.E.K.); 2 Laboratorio Nacional de Celulas-tronco Embrionarias (LaNCE-USP), Departamento de Genetica e Evoluçao, Instituto de Biociencias, Universidade de Sao Paulo, Sao Paulo 05508-060, Brazil; patty_ns8@hotmail.com

**Keywords:** extracellular matrix, arterial aging, smooth muscle cells

## Abstract

Vascular aging is accompanied by the fragmentation of elastic fibers and collagen deposition, leading to reduced distensibility and increased vascular stiffness. A rigid artery facilitates elastin to degradation by MMPs, exposing vascular cells to greater mechanical stress and triggering signaling mechanisms that only exacerbate aging, creating a self-sustaining inflammatory environment that also promotes vascular calcification. In this review, we highlight the role of crosstalk between smooth muscle cells and the vascular extracellular matrix (ECM) and how aging promotes smooth muscle cell phenotypes that ultimately lead to mechanical impairment of aging arteries. Understanding the underlying mechanisms and the role of associated changes in ECM during aging may contribute to new approaches to prevent or delay arterial aging and the onset of cardiovascular diseases.

## 1. Introduction

The extracellular matrix (ECM) is an important component of the vascular wall, allowing it to withstand a wide range of tensile stresses, while preserving its shape and integrity. This is explained by its complex composition, where elastic fibers provide distensibility, while collagens, hyaluronic acid and proteoglycans provide strength and a supporting structure. This network is aided by fibronectin and laminins that, by connecting vascular cells to the ECM, provide the interface for the detection of changes in ECM physical properties [1]. Changes in ECM density, composition and rigidity trigger signaling pathways that, in turn, modulate the cellular ability to synthesize, crosslink and degrade the ECM, shaping it according to homeostatic requirements [2].

The importance of these mechanisms is illustrated by the impact of aging on vascular ECM. After a lifetime of continuous cycles of stretching and recoiling, elastic fibers degenerate, reducing vascular distensibility, and excess collagen deposition accentuates vascular stiffness. These changes are accompanied by increased pulse pressure and myogenic tone, arterial rarefaction, compromised perfusion and damage to organs such as the brain, the kidneys and the retina [3,4,5]. Thus, arterial aging lies at the interface between physiological adaptation and the onset of cardiovascular diseases such as hypertension, heart failure, kidney disease and stroke [6]. In addition, pathological conditions, such as aortic aneurysms, atherosclerosis, diabetes and intimal hyperplasia, exhibit a degree of vascular aging, suggesting that understanding ECM aging may shed light on the pathogenesis of these vascular diseases [7,8,9,10,11].

Here, we discuss the impact of aging on vascular ECM and how the depletion of homeostatic mechanisms that govern ECM composition can influence the mechanical properties and function of large arteries. As the media layer is responsible for the load-bearing capacity of the arteries, we discuss the reciprocal relationship between aging ECM and smooth muscle cells (SMCs) that leads to a range of phenotypes that further exaggerate age-induced vascular changes. Uncovering the essential elements for this crosstalk may give rise to new approaches to prevent or delay age-related vascular pathologies.

## 2. Aging and Vascular Extracellular Matrix Remodeling

### 2.1. Basic Organization of the Vascular Wall

From the inner to the outer surface, the arteries are divided into three basic compartments: intima, media and adventitia. In the intima layer, endothelial cells (ECs) display in its surface heparan sulfate glycosaminoglycans (such as syndecan) and hyaluronic acid, forming a type of surrounding ECM called glycocalyx [12]. This structure extends beyond the cell surface to the vascular lumen, providing the barrier function of ECs that prevents the transmural migration of leukocytes and platelet adhesion [13]. ECs are also supported by a specialized type of ECM called the basement membrane, where networks of type IV collagen and laminins 411 are connected by nidogen and protected against proteolytic degradation by perlecan [14,15]. The basement membrane also sequesters von Willebrand factor, which initiates platelet adhesion and factor VIII-mediated coagulation upon endothelial denudation [15]. The medial layer is the load-bearing unit of the arteries, responsible for their long-range distensibility. In large (elastic) arteries, elastic fibers intervened with concentric sheets of SMCs predominate, surrounded by an incomplete basement membrane [16]. As we move away from the heart this scenario changes with a gradual increase in the prevalence of SMCs over elastic fibers in the muscular arteries, consistent with their control of luminal diameter and blood flow [17]. In contrast, capillaries do not have a media layer; they are composed of pericytes supported by a basement membrane, allowing adequate exchange of gases and fluids [18]. In the adventitia, fibroblasts are incorporated into a loose collagen ECM enriched in hyaluronic acid and its binding partner versican, along with biglycan and decorin (proteoglycans known to bind collagen molecules). In some regions of the arterial tree, the adventitia is where mesenchymal stem cells, vasa vasorum and immune system cells are found [19,20,21,22].

### 2.2. Vascular Aging

**Figure 1:**
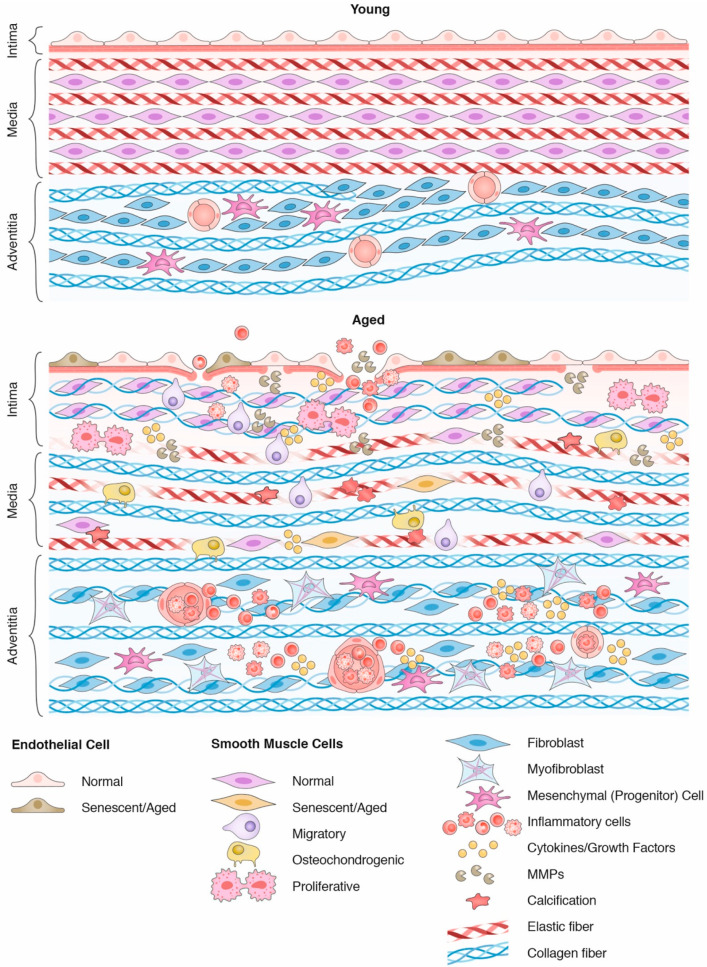
Age-mediated changes in the arterial wall. The upper panel demonstrates the typical features of a young artery, formed by endothelial cells supported by a basement membrane (intima layer), followed by concentric SMC layers in association with elastic fibers, forming lamellar units in the medial layer. Fibroblasts and mesenchymal (progenitor) cells form the adventitial layer along with wavy collagen fibers and vasa vasorum. With aging (bottom panel), endothelial cells become senescent, facilitating the infiltration of inflammatory cells. In the medial layer, elastic fibers become calcified and fragmented, while collagen secretion increases, culminating in vascular stiffening. These changes expose SMC to greater mechanical stress, leading to a broad spectrum of phenotypes that only accentuate arterial aging through the senescence-associated secretory phenotype (SASP). In the adventitia, inflammatory cells use the vasa vasorum to infiltrate the arterial wall, while collagens distend and fibroblasts transdifferentiate into myofibroblasts.

**Figure 2:**
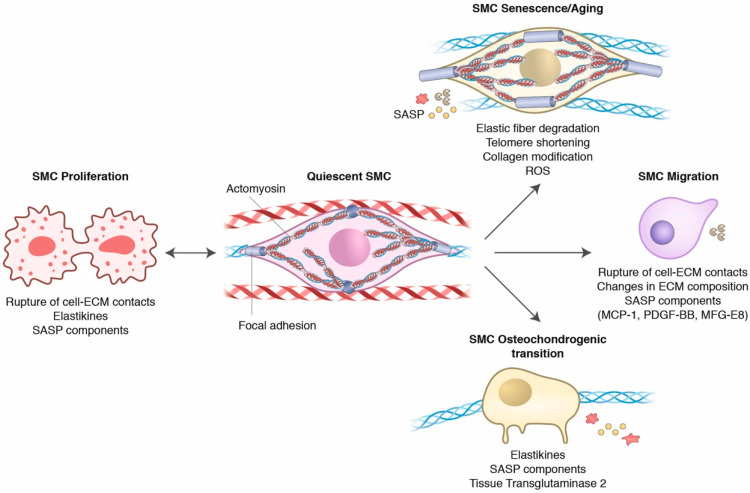
SMC phenotypical changes with aging. Quiescent SMCs are connected to elastic fibers through focal adhesions, modulating actomyosin tension and promoting an ideal mechanical phenotype. Age-induced elastic fiber fragmentation disrupts a signaling pathway that prevents SMC proliferation, providing bioactive peptides (elastikines) that induce SMC proliferation. As elastic fibers protect SMCs from hemodynamic stress, after elastic fiber fragmentation, SMCs stiffen, increasing actin polymerization, focal adhesion size and collagen secretion as a means of dampening the excessive mechanical stress. Collagens surrounding SMCs become progressively glycated, which further reinforces SMC stiffening. Reactive oxygen species (ROS) generated as a response to stress and telomere shortening promote SMC senescence, which is accompanied by the senescence-associated secretory phenotype (SASP), characterized by secretion of cytokines (such as MCP-1), growth factors (PDGFs) and ECM Proteases (MMPs). These factors, together with changes in the microenvironment, favor SMC migration. Changes in ECM and SMC senescence may also favor the osteochondrogenic phenotype, involved in calcification and vascular stiffening.

Aging imposes dramatic changes in the structure and function of arteries with progressive thickening associated with intimal hyperplasia due to the migration of SMCs [23,24] (Figure 1 and Figure 2). These SMCs populate and secrete ECM molecules (including collagens and proteoglycans) in the intima layer (Figure 1) [25] associated to EC phenotypic changes.

ECs are mechanosensors of the cardiovascular system. They line the luminal arterial surface and, thus, are exposed to hemodynamic forces in the form of a frictional dragging force termed shear stress (SS) [26,27]. Laminar SS is a potent protective vascular mechanism against oxidative stress and vascular inflammation, mainly characterized by the synthesis and secretion of Nitric Oxide (NO) in addition to a quiescent EC phenotype [28]. In regions of arterial branching or curvature, this pattern of organized flow is disrupted, decreasing SS and vascular protective properties. Low SS is associated with EC proliferation, along with the expression of inflammatory molecules and increased sensitivity to inflammatory stimuli [29,30,31,32].

A whole set of sensors (called mechanosensors) enables the ECs to detect and transduce flow changes to modulate the cellular response. These sensors are represented by a diverse range of molecules, including ion channels and G-protein-coupled receptors (GPCRs), along with the proteins present at cell structures including the cilia, membrane domains (caveolae and lipid rafts), cell–cell junctions and cell-ECM adhesion complexes [33]. These changes in EC phenotypes and the flow-mediated downstream signaling pathways give rise to biochemical mediators that ultimately modulate vascular function [34,35]. These mediators include the vasodilators NO, prostacyclin and endothelium-derived hyperpolarizing factor, as well as the vasoconstrictors angiotensin II, endothelin and thromboxane A2. Aging affects the compensatory mechanisms underlying these vascular processes, ECs become enlarged and polyploid and, in contrast to young ECs, have reduced regenerative capacity. These changes reduce the availability of NO and increase the synthesis of angiotensin II and endothelin, promoting vasoconstriction [36,37].

In addition to their role in vascular tone, ECs act as guardians of the vascular wall, exhibiting selective permeability to circulating factors (such as cytokines and growth factors) and preventing the infiltration of inflammatory cells. The upregulation of angiotensin II signaling through its cognate receptor (AT1), together with the reduction in NO availability and the consequent increase in reactive oxygen species (ROS) [38], promotes an inflammatory EC phenotype [39,40]. In this scenario, ROS induces telomere shortening [41,42] and mitochondrial DNA damage, activating the mutated ataxia-telangiectasia-CHK2-p53-p21 protein signaling axis, leading to EC senescence. It also activates the activated B-cell nuclear factor κ-light-chain-enhancer (NFκB) in aging ECs [43,44], inducing the expression of adhesion molecules, such as intercellular adhesion molecule (ICAM-1), vascular cell adhesion molecule 1 (VCAM1) and E- and P-Selectins. These inflammatory cell adhesion molecules together with secreted molecules, such as calpain-1, monocyte chemoattractant protein-1 (MCP-1), milk fat globule-EGF factor 8 (MFG-E8), IL-6, IL-8, ROS and necrosis factor tumor-α (TNF-α), prompt the recruitment and adhesion of activated leukocytes (such as neutrophils and monocytes). The migration of these cells through the endothelial barrier is facilitated by the weakening of cell–cell junctions between endothelial cells with aging. The reduced expression of claudin-5, occludin and vascular endothelium cadherin (VE-Cadherin), along with altered distribution of VE-Cadherin and zona-occludens-1 (ZO-1), is accompanied by EC senescence induced by telomere shortening and inflammatory stimuli. These events culminate in loss of junctional integrity [45,46,47,48] that enables leukocytes access to the vascular wall. The leukocytes themselves promote junctional weakening, establishing positive feedback that reinforces vascular inflammatory cell infiltration [49,50].

In addition to changes in the function of ECs and intimal hyperplasia, the media layer also contributes to impairment of the function of the great arteries with aging. The progressive fragmentation of the distensible elastic fibers promotes the stiffening of aging arteries, which is further accentuated by the deposition of stiff collagens [24,51] (Figure 1). The damping properties of the aorta are reduced by these changes. Cyclic changes during each heartbeat are accompanied by aortic distension to store blood and energy during systole, followed by aortic retreat to release blood and energy during diastole, allowing continuous tissue perfusion regardless of intermittent cardiac cycles [52]. This process is hampered by aging-induced aortic stiffening, increasing cardiac work and reducing coronary flow, therefore increasing the risk of cardiovascular disease. Aortic stiffening is translated to increased pulse wave velocity (PWV), an independent predictor of cardiovascular disease [18,53].

Fragmentation of medial elastic fibers exposes the entire artery to increased mechanical stress. In the adventitial layer, this is illustrated by the change in the appearance of collagen fibers from wavy [54,55,56] to distended fibers in elderly individuals [56,57]. Increased stiffness can also release TGF-β from ECM stores, inducing the transdifferentiation of adventitious fibroblasts into myofibroblasts [58], characterized by the expression of α-smooth muscle actin (α-SMA) and desmin [59,60]. This phenotype is associated with increased secretion of collagen types I and III, together with inflammatory cytokines such as MCP-1, which attract monocytes and T cells, reinforcing the inflammatory environment created by the aging of the media and intima layers [61]. 

## 3. Dynamic Crosstalk between Smooth Muscle Cells and the Aged Extracellular Matrix

### 3.1. The Vascular Homeostatic Crosstalk of SMCs and the ECM 

SMCs are responsible for the maintenance and the remodeling of medial ECM, and thus, they are constantly sensing ECM properties, including its composition, density and stiffness. This process (termed mechanosensing) is primarily mediated by cell surface receptors or integrins. Integrins are α-β heterodimers connecting the different ECM molecules to the actin cytoskeleton at focal adhesions [62]. In these sites, stimuli transmitted by integrins reach over 2400 proteins that transform mechanical signal into biochemical intracellular signaling (mechanosignaling) to modulate actomyosin tension, generating a contractile response (termed mechanoresponse) [63,64,65]. It is through actomyosin-based tension that SMC probe mechanical stimuli acting on the ECM, thus establishing a bidirectional mode of mechanosignaling. As cells define a basal mechanical equilibrium with their surroundings, the detection of forces leads to actin cytoskeleton remodeling and reduced or increased actomyosin tension, adjusting the cellular mechanical state to restore vascular mechanical homeostasis [66,67]. The downstream mechanical signaling to alter the cellular genetic program involves the LINC Complex [68], Myocardin-related transcription factors (MRTFs) and YAP/TAZ [69,70,71]. By modulating the activity of these transcription factors, SMCs promote ECM remodeling either by the de novo synthesis of ECM molecules or by the expression and secretion of ECM proteases [72,73,74,75]. Furthermore, the signals originated from the vascular ECM support the quiescent differentiated phenotype of the SMC, thus preventing migration and proliferation events beyond what is necessary for vascular renewal [76,77,78].

### 3.2. Aging Influences on SMC and ECM Crosstalk

The first sign of aging in the vasculature is the loss of distensibility of the aortic arch and the increase in the thickness of the conduit arteries [79,80]. Vascular distensibility is conferred by elastic fibers, structures established early during embryonic development, where SMCs secrete and organize elastin and microfibrils through the processes of coacervation (a two-step temperature-sensitive process in which soluble tropoelastin molecules aggregate to form oligomers that then associate to form large elastic polymers), lysyl oxidase-mediated crosslinking and lateral association [81,82]. After a lifetime of mechanical deformation, elastic fibers fragment as a consequence of the combination of mechanical fatigue, calcification, non-enzymatic glycation and enzymatic degradation [25,83,84,85]. Consequently, there is an increase in collagen content and crosslinking by enzymatic (via lysyl oxidases, LOX) and non-enzymatic (by advanced glycation end products, AGEs) mechanisms, further increasing vascular stiffness [86,87,88]. Increased vascular stiffness is detected and transmitted by cell surface integrins at focal adhesions [67]. Stiffening of the ECM leads to conformational changes in integrins, culminating in activation and clustering. When active, integrins promote talin unfolding, creating a binding site for vinculin, which reinforces focal adhesions by increasing the interactios between talin and the actin cytoskeleton [89]. These conformational changes also activate kinases (such as the focal adhesion kinase—FAK), leading to the recruitment of additional kinases, adapters and accessory proteins, leading to centripetal focal adhesion growth, and finally allowing SMC to adjust actomyosin-based tension to counteract increased stiffness, thus restoring mechanical homeostasis [65,66,67]. In response to increased collagen and ECM rigidity, aged SMCs increase the expression of β_1_-integrin [90], known to form collagen receptors after dimerization with α_1_-, α_2_- and α_v_-integrins [62]. Using atomic force microscopy (AFM), it has been shown that, when compared to SMCs from young animals, aged SMCs are stiffer, a property that is dependent on the actin cytoskeleton in the cellular cortex [90,91]. As the actin cytoskeleton is connected to focal adhesions, it was also observed that aged SMCs exhibit increased adhesion strength (measured as the force required to break the bond between the ECM-coated probe and the cell) [90]. This evidence is supported by studies showing an increase in actin fiber content and focal adhesion size (Figure 2) in aged SMCs dependent on Rho kinase (ROCK) activation, a key step for actomyosin modulation [92]. However, these signals are not translated into efficient actomyosin tension. Instead, aged SMCs are unable to remodel a collagen-based ECM [79,92], suggesting that in close resemblance to the ECM, aged SMCs lose a component of distensibility with aging. This can be explained by reduced vinculin expression and FAK activation [92,93], which may limit the ability of the SMC to transduce and respond to changes in ECM properties [79,92]. This impairment is translated into prevention of the vasoconstrictor response of the skeletal muscle feeding arteries [92], inability to contract a collagenous ECM and reduced relaxation in the aortic rings [79].

### 3.3. Elastic Fiber Fragmentation and SMC Phenotypical Changes

In addition to the feed-forwarding mechanism that induces collagen secretion and increased stiffness, elastic fiber fragmentation with aging is known to release elastin-derived bioactive peptides called elastikines [94]. These peptides bind to and activate the elastin receptor complex [95,96], a heterotrimer formed by elastin binding protein (EBP), associated with protective protein/cathepsin A (PPCA) and membrane-associated neuraminidase-1 (Neu-1). The binding of elastikines to EBP promotes the activation of phospholipase C (PLC) and protein kinase C (PKC), culminating in a rapid increase in intracellular calcium concentration and activation of the FAK-Src-ERK signaling axis. As a consequence, there is an accumulation of cyclins and cyclin-dependent kinases (CDKs) that ultimately promote the proliferation of SMCs [97] (Figure 2). 

Under physiological conditions, SMCs display the so-called contractile phenotype, characterized by the expression of contractile genes (such as α-SMA, SM-22α, MYH11, calponin and smoothelin) and discrete secretory and proliferative activities [98]. The proliferative action of elastikines suggests that they switch SMC from a contractile to a proliferative/synthetic phenotype. In fact, aged SMCs appear morphologically irregular and enriched in endoplasmic reticulum, Golgi apparatus and free ribosomes, characteristics of a synthetic phenotype [80,99]. Furthermore, it has been shown that elastikines can induce the osteochondrogenic transition of SMCs [100] (Figure 2), characterized by cell biomineralization (calcification), reduced contractile gene expression [101,102] and increased levels of mineralization markers, such as alkaline phosphatase, type II and type X collagens, runt-related transcription factor 2 (RUNX2) and SRY-Box 9 transcription factor (SOX9) [101,103,104]. There is evidence to suggest that these changes are dependent on the activation of calpain-1 and tissue transglutaminase-2 (TG2). Calpain-1 activity increases calcified sites in the human aorta, and its overexpression promotes elastin degradation, decreases the expression of osteopontin and osteonectin (calcification inhibitors), while promoting senescence and increasing activity of alkaline phosphatase and type-I membrane MMP (MT1-MMP) in young SMCs [105]. Likewise, TG2 activation is increased with arterial aging [106,107], playing a role in collagen crosslinking and turnover reduction. This increase was also observed in the osteochondrogenic transition of SMCs, where TG2 stimulated the expression of RUNX2 and BMP-2 (Bone morphogenic protein-2), while suppressing the expression of osteopontin [108,109].

### 3.4. ECM Changes and the Activation of TGF-β Signaling in SMCs

Elastic fibers (the primary target of MMP-2 and MMP-9) are protected by microfibrils, support structures based on the polymerization of proteins called fibrillins in a bead-on-a-string configuration [81]. These polymers associate laterally with each other and after the addition of latent TGF-β binding proteins (LTBPs), microfibril-associated proteins, microfibril-associated glycoproteins, type VIII collagen, emilins, proteoglycans, glycosaminoglycan heparan sulfate and isoforms of fibulin provides a support network for elastic fibers [110,111,112,113]. Furthermore, through the action of LTBPs, microfibrils sequester growth factors, such as transforming growth factor-β (TGF-β), thus controlling their bioavailability and activation [114]. Proteolytic events in elastic fibers can give MMPs access to cleave LTBPs, releasing TGF-β molecules that bind and activate their cognate receptors on SMCs. TGF-β signals mainly through SMADs, cytoplasmic proteins that, when active, translocate to the nucleus and increase the expression of genes such as fibronectin, type I–III collagens and plasminogen activator inhibitor-1 (PAI-1) and the MMP inhibitors (tissue inhibitor metalloproteinases—TIMPs) in aged SMCs [115,116]. This idea is supported by the findings that PAI-1 activity and expression increase in experimental models of aging and in elderly individuals [117,118,119]. As TGF-β is also known to promote SMC contractile gene expression and cell cycle arrest [98,120,121], aging can progressively uncouple TGF-β control over the cell cycle from ECM secretion in SMCs, as seen in the context of atherosclerosis [122]. This is illustrated by the increased expression of TGF-β by rat SMCs, as well as by the findings that TGF-β increases the synthesis and secretion of ECM molecules and growth factors in SMCs, despite increased TGF-β processing, reduced availability of the receptor and being unable to inhibit cell cycle progression [123]. This uncoupling response may favor the osteochondrogenic phenotype and, therefore, the migration and proliferation of SMCs. Given the same findings, it was suggested that SMCs can be selected by unknown mechanisms, favoring the survival of those resistant to TGF-β. This would explain the observations of apoptotic events and reduction in the number of SMCs [124,125], as well as the phenotype of senescent polyploid SMCs during vascular aging [126,127,128,129].

## 4. Cellular Senescence in Aging

Cellular senescence has been shown to be an important contributor to aging and understanding this process can guide therapies to minimize aging-related diseases. Although aging is a progressive loss of function over time, cell senescence can occur at any point in cell life. Senescent cells are eliminated by the immune system. The impairment of these mechanisms leads to the accumulation of senescent cells, which release factors that can promote or exacerbate tissue damage.

The concept of cell senescence was first postulated by Hayflick and Moorehead, who observed that primary cells could only proliferate up to a certain limit (the Hayflick limit), determined by telomere length [130,131]. After telomere shortage and dysfunction, the DNA damage response is triggered, activating p16 and p53 [132]. Active p16 inhibits cyclin D-CDK4/6, whereas p53 induces the expression of the cyclin-dependent kinase (CDK) inhibitor p21, suppressing the activity of cyclin E-CDK2 [131,133]. Furthermore, p16 and p21 support the active and hypophosphorylated state of the retinoblastoma protein (pRB), inhibiting E2F function [108] and, thus, disrupting the expression of genes responsible for the mitotic division of SMCs [134].

### 4.1. Collagen in Promotion of SMC Senescence

The influence of vascular ECM on cell senescence process has been elegantly demonstrated by studies in mice bearing targeted substitutions in the Col1a1 gene (Col1a1^r/r^) [135]. These mice carry three substitutions in the collagen triplet sequences: the residues of glutamine 774 and alanine 777 are replaced by proline residues, while the residue of isoleucine 776 is replaced by a methionine. As these residues flank the collagenase recognition site (lying between residues 775–776), these changes prevent collagenase-mediated degradation, in addition to promote collagen helix stabilization [136,137]. The authors found that Col1a1^r/r^ SMCs were susceptible to angiotensin II-induced senescence, denoted by positive β-galactosidase staining and increased expression of p16 and p21 [135]. To directly test the relevance to SMC senescence, they cultured human aortic SMCs in the presence of tail collagen isolated from wild-type mice or Col1a1^r/r^ and found that the mutated collagen reduced the doubling of the SMC population and induced an early increase in staining of β-galactosidase in aged serum-deprived cultures, in association with premature upregulation of p16 and p21 and changes in SMC morphology [135]. The underlying mechanism has been attributed to the need for collagen processing to activate specific integrin heterodimers. Although type I collagen is recognized by α_1_β_1_ and α_1_β_2_ integrins [138,139], it is only upon collagenase cleavage that cryptic RGD sites are exposed to engage with α_v_β_3_ integrins [140,141]. Thus, it was postulated that the Col1a1^r/r^ mutation impairs α_v_β_3_ integrin signaling. In fact, antagonism of α_v_β_3_ integrins with echistatin or LM609 (α_v_β_3_ integrin blocking antibody) in wild-type SMCs reproduced the senescent phenotype of SMCs grown on Col1a1^r/r^ collagen [135]. Finally, by using vitronectin (agonist of α_v_β_3_-integrin agonist), the senescence induced by Col1a1^r/r^ collagen was abolished [135], demonstrating that under physiological conditions, SMCs can induce low-level collagen proteolysis, culminating in autocrine and/or paracrine activation of α_v_β_3_ integrin signaling, thus sustaining SMC longevity. In addition, these findings highlight the relevance of AGE-dependent collagen crosslinking for SMC aging, as it promotes a similar impairment in ECM remodeling, protecting collagens from collagenase-mediated degradation during arterial aging [86,87,88].

### 4.2. The Senescence-Associated Secretory Phenotype (SASP)

Similar to fibroblasts, aged SMCs exhibit the so-called senescence-associated secretory phenotype (SASP) [142,143] (Figure 2) characterized by the secretion of large amounts of chemoattractant (such as MCP-1, macrophage inflammatory protein-1α (MIP-1α)**/**CCL3 and MIP-1β/CCL4), cytokines (interleukin (IL) -1, IL-6 and IL-8), ECM proteases (MMP-2 and MMP-9) and growth factors (such as the platelet-derived growth factor-BB (PDGF-BB), granulocyte colony-stimulating factor (G-CSF) and basic fibroblast growth factor (bFGF)) [144,145,146,147,148,149]. This secretory phenotype has autocrine and paracrine effects. Autocrine effects are observed as the migratory response of aged SMCs (Figure 2) [150] in response to MCP-1, allowing them to populate (in a PDGF-BB-dependent manner [151,152]) and induce a fibrotic response in the intimal layer [153,154]. A putative upstream molecule for this MCP-1-dependent phenomenon is MFG-E8, which increases in response to angiotensin II upregulation in arterial aging [155]. It is a α_v_β_5_-integrin ligand that triggers ERK 1/2 activation, leading to increased PDGF/PDGFR signaling and accumulation of CDK-4 and PCNA, ultimately promoting SMC proliferation [156]. In aged SMCs, MGF-E8 induces NF_k_B activation and the synthesis and secretion of MCP-1, MMP-2 and MMP-9 [157], key components of SASP [142]. Its silencing prevents the invasive phenotype of SMCs, in an MCP-1-dependent manner, since the treatment of SMCs with vCCl (a blocker for the MCP-1 CCR2 receptor) inhibits SMC invasiveness in the presence of MFG-E8. These findings suggest that MFG-E8-α_v_β_5_-integrins act upstream of MCP-1-induced SMC migration and proliferation (Figure 2) and highlight the potential to block this signaling pathway in preventing intimal thickening of human arterial aging. Interestingly, serum MFG-E8 correlates with the human arterial stiffness index, PWV [158].

The paracrine effects of SASP establish a feed-forward loop that further accentuates vascular aging by priming adjacent macrophages, endothelial and SMC towards an inflammatory phenotype. In conjunction with MIP-1α and MIP-1β, MCP-1 recruits monocytes and macrophages to the vascular wall. This event is associated with a burst of inflammatory mediators, oxidative species (such as ROS and reactive nitrogen species [RNS]), arachidonic metabolites, vasoactive polyamines, cytokines, chemokines and ECM-degrading enzymes [159]. These molecules attract other inflammatory cells, reinforcing the establishment of an oxidative inflammatory environment. Oxidative species, such as hypochlorous acid and peroxynitrite, can induce cysteine switch, a molecular mechanism that resumes the latency of ECM-degrading enzymes of the MMP family [160]. MMPs are versatile enzymes, capable of degrading virtually any ECM molecule [161]. They are represented by 23 soluble or membrane-bound enzymes, secreted as zymogens that, after proteolytic cleavage or ROS-mediated thiol oxidation, become active in the extracellular space [162,163,164]. Aging increases the presence of MMP-2, MMP-7, MMP-9 and MMP-14 in the arterial wall [25,165,166], while reducing TIMPs [51,163]. MMP-14 is a membrane-type MMP that cleaves and activates pro-MMP-2 [165]. MMP-2, MMP-7 and MMP-9 act as both elastases that accentuate the degradation of elastic fibers, disrupting the internal elastic lamina and allowing intimal invasion by SMCs [51,167,168,169], and as nonspecific proteases, degrading collagen, fibronectin and laminin. As a consequence, the adverse remodeling of the vascular ECM is reinforced, changing the EC and SMC phenotype to a more secretory, migratory, proliferative and senescent phenotype, thus contributing to vascular fibrosis and increased intima-media thickness, further increasing arterial stiffness [105,170]. Indeed, exposure of young animals to angiotensin II leads to a phenotype that resembles arterial aging, characterized by fibrosis, MMP-2 activation and increased intima-media thickness [114]. On the other hand, MMP inhibition with PD166793 blunted age-associated vascular fibrosis and remodeling in experimental models [171,172,173]. 

## 5. Perspectives

As aging and arterial stiffness are at the interface between physiological adaptation and the onset of cardiovascular disease, understanding the signaling mechanisms underlying aging SMC phenotypes would shed light on new approaches to prevent or delay arterial stiffness and improve cardiovascular health. It was recently showed that Senolytics (desatinib plus quercetin) decrease senescent cells in humans, declining plasma levels of key SASP factors, including IL-1α, -2, -6 and -9 and MMP-2, -9 and -12 [174]. In addition, metformin and resveratrol have been proposed to combat aging and aging-related diseases, although the mechanisms underlying their effect in promoting genome protection and preventing oxidative stress remain to be elucidated [175,176]. In agreement with the proposed role for SMC in arterial aging, the current therapies for vascular stiffness include diuretics and/or calcium-channel blockers associated with angiotensin II blockade, targeting signaling pathways in control of actomyosin-based tension in SMCs [177].

Vascular calcification accompanies aging and atherosclerosis, but hitherto, it has not been a direct therapeutic target [178]. In this context, ECM-focused therapies may be explored to expand ongoing efforts limited to skin aging [179]. 

In this review, we highlight the active role of environmental sensing by SMCs and its relation with the ECM to affect arterial aging. In this scenario, elastic fiber degradation and undamped exposure to hemodynamic factors trigger signaling pathways in the promotion of a broad spectrum of SMC phenotypes that ultimately cause intimal hyperplasia, collagen deposition and the establishment of a constant low-level state of inflammation contributing to the aging on the vascular wall (Figure 1). The cellular and molecular mechanisms that lead to the development of heterogeneous SMC phenotypes in aging arteries are poorly understood and will be critical to design therapeutic strategies to prevent vascular deterioration and the onset of cardiovascular derangements.

We postulate that the gradual elastic fiber fragmentation and increased exposure to hemodynamic forces gradually increase the stiffness of SMCs over time, leading to collagen secretion. As collagen is known to promote cell proliferation [180], this is consistent with the proliferation and replicative senescence of SMC [126,127,128,151]. Senescent SMC exhibits the secretory phenotype (SASP) [142] and microRNA-203 [181] upregulation may decrease the expression of structural elements such as vinculin and partially blunt SMC viscoelasticity (culminating in ROCK-mediated increased stiffness, but reduction of contractility) and the ability to regenerate their mechanosensitive pathways [79,92]. This would prepare cells for the osteochondrogenic phenotype, as mesenchymal cells differentiate into osteocytes depending on collagen type I, ROCK activity and ECM stiffness [182,183]. Interestingly, inhibition of ROCK-mediated myosin phosphorylation with blebbistatin prevents the differentiation of mesenchymal cells into osteocytes [183], raising the possibility that this drug can be used to prevent SMC stiffening and the osteochondrogenic phenotype. As SMCs use ROCK-dependent signaling pathways to migrate [184], finding a way to target ROCK in rigid cells would, therefore, limit intimal hyperplasia, vascular calcification and the feedback loop in the inflammatory response elicited by SMCs.

In summary, we highlight the essential role of the dynamic vascular ECM and SMCs crosstalk mechanisms in aging and pathologic states. The osteochondrogenic and senescence-associated secretory phenotypes of senescent SMCs is amplified by the age-induced changes in ECM, establishing a fertile inflammatory and oxidative environment predisposing to development of age-related cardiovascular pathologies. Population aging has several unmet clinical needs and represents an opportunity to develop new approaches to delay SMC aging and age-related vascular derangement based on improved understanding of these vascular biology processes.
